# Supplementary material for: Genome-Wide Association Study of Varenicline-Aided Smoking Cessation
Source: Nicotine Tob Res. 2025 Jan 10;27(10):1684–94. doi: 10.1093/ntr/ntaf009 (PMC12453674; doi:10.1093/ntr/ntaf009)
Supplement: ntaf009_suppl_Supplementary_Material [file ntaf009_suppl_supplementary_material.pdf]

Genome-wide association study of varenicline-aided smoking cessation

Kayesha Coley PhD[1*], Qingning Wang PhD[1], Richard Packer PhD[1,2], Catherine John PhD[1,2], Erik Abner PhD[3], Kadri Reis PhD[3], Estonian Biobank Research Team[3], Khaled F. Bedair PhD[4], Sundararajan Srinivasan PhD[4], Sara Paciga PhD[5], Craig Hyde PhD[5], Robert C. Free PhD[2,6], Nicola F. Reeve PhD[1], David J. Shepherd BM BCh[1], Tõnu Esko PhD[3,7], Colin Palmer PhD[4], Ewan Pearson PhD[4], Anders Malarstig PhD[8,9], Martin D. Tobin PhD[1,2], Chiara Batini PhD[1,2*]

[1]Department of Population Health Sciences, University of Leicester, Leicester, UK

[2]University Hospitals of Leicester NHS Trust, Groby Road, Leicester, UK

[3]Estonian Genome Centre, Institute of Genomics, University of Tartu, Tartu, Estonia

[4]Pat Macpherson Centre for Pharmacogenetics and Pharmacogenomics, Division of Population Health and Genomics, Ninewells Hospital and Medical School, University of Dundee, Dundee, UK

[5]Pfizer Worldwide Research, Development and Medical, USA

[6]School of Computing and Mathematical Sciences, University of Leicester, Leicester, UK

[7]Program in Medical and Population Genetics, The Broad Institute of MIT and Harvard, Cambridge, MA, USA

[8]Department of Medical Epidemiology and Biostatistics, Karolinska Institute, Stockholm, Sweden

[9]Pfizer Worldwide Research, Development and Medical, Stockholm, Sweden

[*]Correspondence to KC (kc330@leicester.ac.uk; +44 (0)116 229 7251) and CB (cb334@leicester.ac.uk; +44 (0)116 229 7251)

Supplementary Material

Supplementary Text

Stage 1 cohorts

Estonian Biobank[1] is a population-based cohort with deep baseline phenotyping and clinical events for over 200,000 participants aged ≥18 years from Estonia. In this study, data freeze (2020v4) was used. All biobank participants have signed a broad informed consent form and information on ICD-10 codes is obtained via regular linking with the national Health Insurance Fund and other relevant databases, with majority of the electronic health records having been collected since 2004.

The Extended Cohort for E-health, Environment and DNA (EXCEED) Study[2] is a cohort of approximately 10,000 participants (aged between 40 and 69 years) recruited from Leicester, Leicestershire and Rutland, mainly through local general practices since 2013.

Genetics of Diabetes Audit and Research in Tayside Scotland (GoDARTS)[3] is a cohort study of over 16,000 individuals, approximately 50% of which have type II diabetes, from the Tayside region of Scotland, and includes lifestyle data, biomarker measurements and medical records.

Genetics of the Scottish Health Research Register (GoSHARE)[4] is a sub-study of the SHARE initiative where almost 75,000 individuals living in Scotland have linked EHRs and consented for genotyping analysis.

UK Biobank[5] is a cohort of approximately 500,000 individuals recruited from across the United Kingdom. Individuals aged between 40 and 69 years were recruited from the general population between 2006 and 2010.

Supplementary Figures

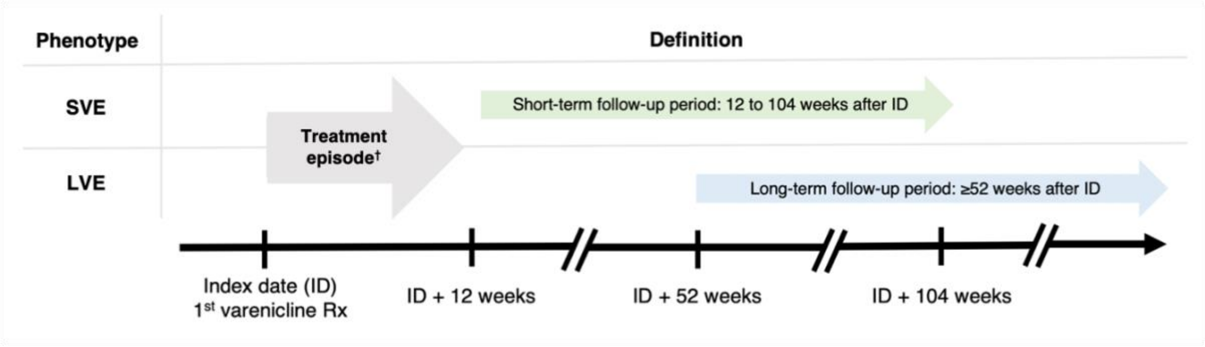

Supplementary Figure 1 Description of phenotype definitions for SVE and LVE defined in cohort studies. †Based on a standard 12-week treatment plan. *Abbreviations:* Rx, prescription.

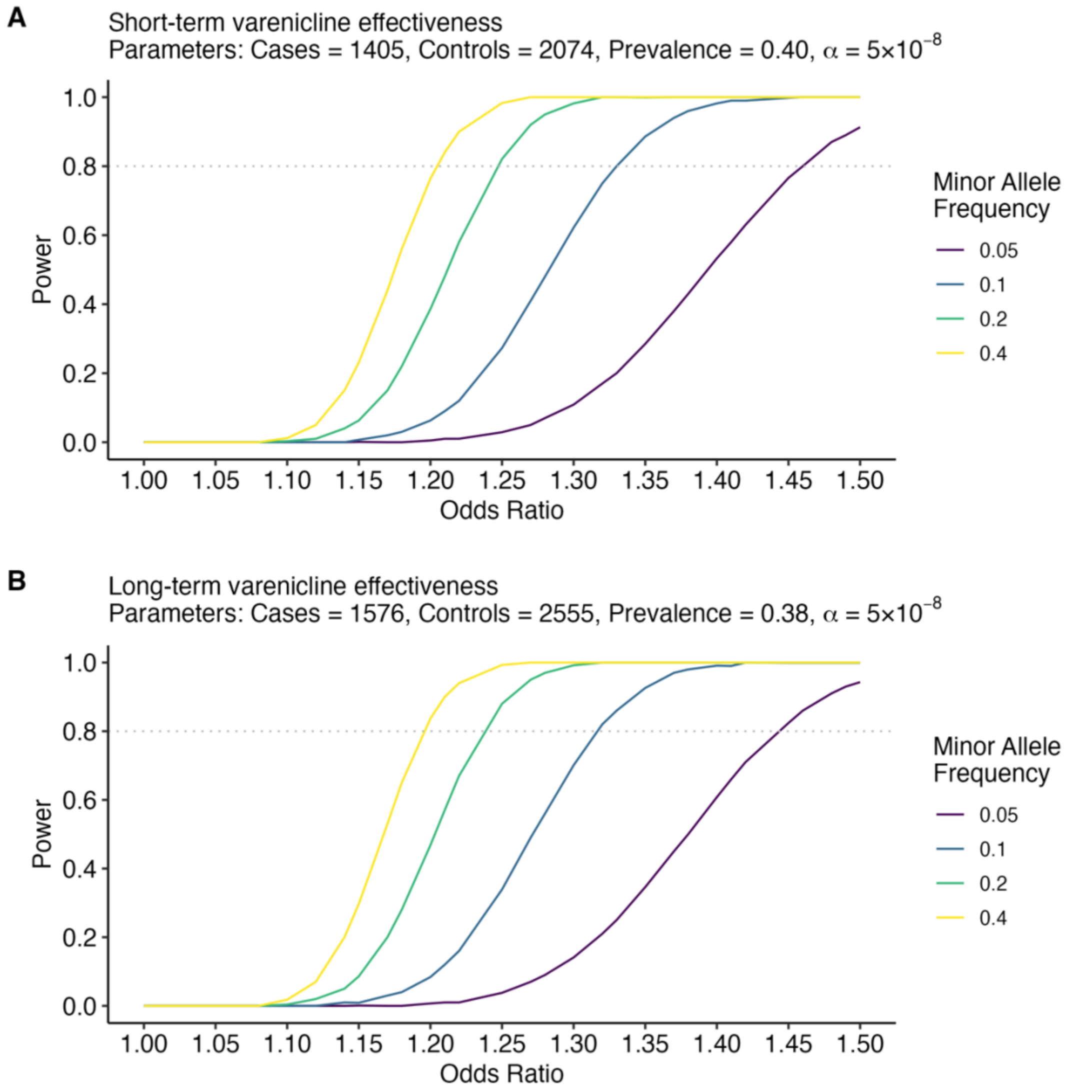

Supplementary Figure 2 Power-odds ratio curves for Stage 1 GWASs of **(A)** SVE and **(B)** LVE.

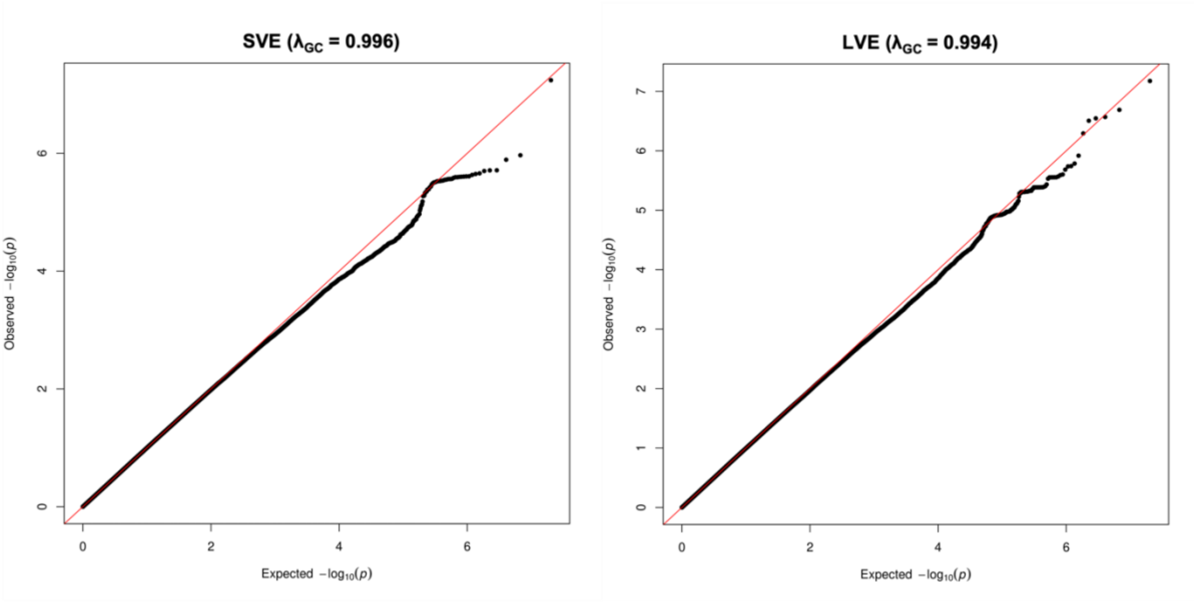

Supplementary Figure 3 QQ plots for Stage 1 GWASs of short-term varenicline effectiveness (SVE) and long-term varenicline effectiveness (LVE). λ_{GC}, genomic control lambda.

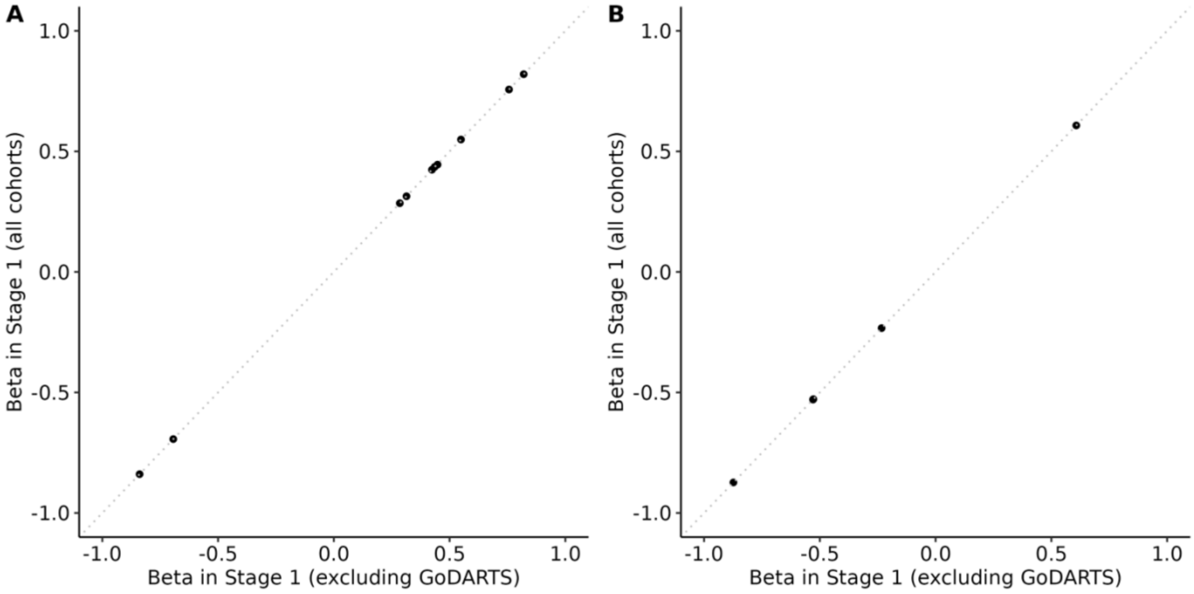

Supplementary Figure 4 Stage 1 effect size (beta) comparison of **(A)** SVE and **(B)** LVE sentinels when including all cohorts, compared to excluding GoDARTS.

Supplementary Tables

Supplementary Table 1 Read v2 and Read v3 (CTV3) codes used to define smoking status.

Code		Description	Inferred smoking status
Read v2	Read v3		
1371.	XE0oh	Never smoked tobacco	Non-smoker
	Ub0oq	Non-smoker	Non-smoker
1372.	XE0oi	Trivial smoker - < 1 cig/day	Smoker
	Ub1tR	Occasional cigarette smoker	Smoker
1373.	1373.	Light smoker - 1-9 cigs/day	Smoker
1374.	1374.	Moderate smoker - 10-19 cigs/d	Smoker
1375.	1375.	Heavy smoker - 20-39 cigs/day	Smoker
1376.	1376.	Very heavy smoker - 40+cigs/d	Smoker
1377.	1377.	Ex-trivial smoker (<1/day)	Non-smoker
1378.	1378.	Ex-light smoker (1-9/day)	Non-smoker
1379.	1379.	Ex-moderate smoker (10-19/day)	Non-smoker
137A.	137A.	Ex-heavy smoker (20-39/day)	Non-smoker
137B.	137B.	Ex-very heavy smoker (40+/day)	Non-smoker
137e.	XaBSp	Smoking restarted	Smoker
137F.	137F.	Ex-smoker - amount unknown	Non-smoker
137H.	137H.	Pipe smoker	Smoker
137j.	Xa1bv	Ex-cigarette smoker	Non-smoker
137J.	137J.	Cigar smoker	Smoker
137K.	137K.	Stopped smoking	Non-Smoker
137K0	XaQzw	Recently stopped smoking	Non-smoker
137l.	XaQ8V	Ex roll-up cigarette smoker	Non-smoker
137m.	XaWNE	Failed attempt to stop smoking	Smoker
137L.	137L.	Current non-smoker	Non-smoker
137N.	137N.	Ex pipe smoker	Non-smoker
137O.	137O.	Ex cigar smoker	Non-smoker
137P.	XE0oq	Cigarette smoker	Smoker
137R.	137R.	Current smoker	Smoker
137S.	Ub1na	Ex-smoker	Non-smoker

Supplementary Table 2 Characteristics of **(A)** participating cohorts in the Stage 1 GWASs of varenicline effectiveness phenotypes, and **(B)** the Stage 2 dataset of comparable randomised controlled trial (RCT) endpoints.

	**(A) Stage 1**					**(B) Stage 2**
**Cohort**	**Estonian Biobank**	**EXCEED**	**GoDARTS**	**GoSHARE**	**UK Biobank**	**Pfizer**
**Recruitment country**	Estonia	UK	UK (Scotland)	UK (Scotland)	UK	Worldwide
**Phenotype data**	EHRs and questionnaire data*	EHRs	EHRs	EHRs	EHRs	RCTs
**Genomic data‡**						
**Genotyping array**	Illumina Global Screening v1.0 and v2.0	Applied Biosystems UK Biobank Axiom	Affymetrix SNP 6.0; Illumina HumanOmniExpress; Illumina Infinium Broad†	Illumina Global Screening v2.0	Applied Biosystems UK BiLEVE Axiom; Applied Biosystems UK Biobank Axiom	Illumina OmniExpressExome Array
**Pre-imputation sample filters**	Call rate ≥95%	Call rate ≥97%	Call rate ≥95%	Call rate ≥95%	Call rate ≥95%	Call rate ≥99%
**Pre-imputation variant filters**	Call rate ≥95%<br>HWE *p*-value $\geq 1\times10^{-4}$<br>MAF ≥0.01	Call rate ≥95%<br>HWE *p*-value $>1\times10^{-6}$<br>MAF >0.01	Call rate ≥97%<br>HWE *p*-value $>1\times10^{-6}$<br>MAF >0.01	Call rate ≥97%<br>HWE *p*-value $>1\times10^{-6}$<br>MAF >0.01	Call rate ≥90%<br>HWE *p*-value $>1\times10^{-12}$<br>MAF >0.0001	Call rate ≥95%
**Imputation panel**	Estonian Reference Panel	TOPMed	HRC	HRC	HRC and UK10K + 1000 Genomes phase 3	1000 Genomes phase 1 (version 3)
**Post-imputation variant filters**	Imputation quality ≥0.3; MAF ≥1%; MAC ≥20					Imputation quality ≥0.3; MAF ≥1%; MAC ≥20; AF difference <0.2 compared to 1000 Genomes (Europeans)
**Association testing**						
**Software**	SAIGE v0.43.1	PLINK 2.0	PLINK 2.0	SNPTEST v2.5.4-beta3	PLINK 2.0	ProbABEL
**Model**	Logistic mixed model	Firth-fallback logistic regression	Firth-fallback logistic regression	Frequentist	Firth-fallback logistic regression	Logistic regression
**Related individuals**	Included	Excluded	Excluded	Excluded	Excluded	Excluded
**Covariates**	Age, age², sex, PCs 1-10	Age, age², sex, PCs 1-10	Age, age², sex, PCs 1-5	Age, age², sex, PCs 1-10	Age, age², sex, genotyping array, PCs 1-10	Age, sex, source protocol, PCs 1-4

[*]Varenicline effectiveness phenotypes were defined using a combination of primary care EHRs (to obtain varenicline prescription events) and baseline questionnaire data (to obtain smoking status). [†]In GoDARTS, association testing was performed in individuals genotyped on each of the three different separately, and subsequently meta-analysed. [‡]Further description of the sample-level and variant-level genotyping quality control performed by individual cohort studies are available elsewhere for Estonian Biobank (Laisk *et al*[6]), EXCEED (Williams *et al*[7]), GoDARTS and GoSHARE (Srinivasan *et al*[8]) and UK Biobank (Bycroft *et al*[5]). *Abbreviations:* AF, allele frequency; EHR, electronic health record; HRC, Haplotype Reference Consortium; HWE, Hardy-Weinberg equilibrium; MAC, minor allele count; MAF, minor allele frequency; PC, principal component; TOPMed, Trans-Omics for Precision Medicine.

Supplementary Table 3 Sentinel variants associated with **(A)** SVE and **(B)** LVE in Stage 1 GWASs.

Variant	Chr:Position (hg19)	Info score*	Direction†	Heterogeneity estimate, I^2 (%)
**(A) Short-term varenicline effectiveness (SVE)**				
rs186444103	2:40054022	0.982	???++	0
rs4364036	2:162904624	0.900	????+	0
rs4686373	3:9853437	0.998	?+?-+	49.9
rs78597169	8:117830288	0.891	???--	0
rs2297038	10:30663464	0.817	?+??+	0
rs9600669	13:76776621	0.987	?-+++	43.0
rs8003262	14:102050501	0.980	???++	0
rs149318872	15:101162067	0.753	????-	0
rs10421326	19:473572	0.861	????+	0
rs827937	20:46793101	0.991	?+??+	0
**(B) Long-term varenicline effectiveness (LVE)**				
rs895545	2:121721467	1.000	???--	0
rs7712227	5:118656769	0.978	?+?--	0
rs78695099	6:100195347	0.927	????-	0
rs35443123	7:85173800	0.990	--??-	8.2
rs766169	22:47680100	1.000	???++	0

*Info score calculated in UK Biobank. †Direction column has the following order: Estonian Biobank, EXCEED, GoDARTS, GoSHARE, UK Biobank. *Abbreviations:* Chr, chromosome.

Supplementary Table 4 95% credible sets generated by fine-mapping of loci associated with **(A)** SVE and **(B)** LVE in Stage 1.

Locus sentinel	Chr:Position (hg19)	Variant ID	Effect allele	Other allele	MAF	*N*	OR [95% CI]	*p*-value	PIP
**(A) Short-term varenicline effectiveness (SVE)**									
rs186444103	2:40054022	rs186444103	T	G	0.026	3023	2.27 [1.61, 3.21]	3.20E-06	0.3347
rs186444103	2:40027750	rs74900673	T	C	0.025	3023	2.26 [1.60, 3.18]	3.74E-06	0.3113
rs186444103	2:40062149	rs115068901	T	C	0.025	3023	2.27 [1.61, 3.22]	3.67E-06	0.3001
rs186444103	2:40126792	rs114238370	T	C	0.022	3023	2.18 [1.49, 3.18]	5.37E-05	0.0539
rs4364036	2:162904624	rs4364036	T	C	0.034	2699	2.13 [1.55, 2.92]	2.55E-06	0.6548
rs4364036	2:162869850	rs142456029	A	G	0.023	2699	2.47 [1.69, 3.62]	3.28E-06	0.2289
rs4364036	2:162818274	rs79187915	A	G	0.030	2699	1.98 [1.42, 2.76]	5.55E-05	0.0918
rs4686373	3:9853437	rs4686373	A	G	0.090	3167	1.55 [1.29, 1.85]	2.50E-06	0.5511
rs4686373	3:9809082	rs293796	T	C	0.090	3167	1.49 [1.25, 1.79]	1.20E-05	0.1642
rs4686373	3:9852059	rs34761997	CGAT	C	0.110	2699	0.71 [0.59, 0.84]	1.11E-04	0.0294
rs4686373	3:9842742	rs6786980	A	G	0.112	3375	0.73 [0.62, 0.86]	1.49E-04	0.0255
rs4686373	3:9865173	3:9865173:G:GT	G	GT	0.109	2699	0.71 [0.59, 0.85]	1.33E-04	0.0254
rs4686373	3:9867093	rs6443268	C	G	0.111	3375	1.36 [1.15, 1.60]	2.27E-04	0.0181
rs4686373	3:9856892	rs9836708	T	C	0.111	3375	1.36 [1.15, 1.60]	2.45E-04	0.0171
rs4686373	3:9858891	rs58647240	C	G	0.111	3375	1.36 [1.15, 1.60]	2.49E-04	0.0168
rs4686373	3:9864641	rs6766418	A	C	0.111	3375	1.36 [1.15, 1.60]	2.52E-04	0.0167
rs4686373	3:9864272	rs7623315	A	G	0.111	3375	0.74 [0.63, 0.87]	2.54E-04	0.0166
rs4686373	3:9857371	rs7610410	A	T	0.111	3375	0.74 [0.63, 0.87]	2.56E-04	0.0165
rs4686373	3:9857514	rs7652460	A	G	0.111	3375	1.36 [1.15, 1.60]	2.56E-04	0.0165
rs4686373	3:9866064	rs4686376	C	G	0.111	3375	1.36 [1.15, 1.60]	2.58E-04	0.0162
rs4686373	3:9861053	rs3806666	T	C	0.111	3375	1.35 [1.15, 1.59]	2.70E-04	0.0158
rs4686373	3:10513705	rs12637358	C	G	0.312	2765	0.80 [0.71, 0.91]	3.25E-04	0.0156
rs78597169	8:117830288	rs78597169	A	T	0.026	3023	0.43 [0.30, 0.61]	3.12E-06	0.5825
rs78597169	8:118358227	rs753006634	A	AT	0.382	2699	0.8 [0.70, 0.90]	4.34E-04	0.1771
rs78597169	8:117841402	rs56777536	T	C	0.196	3441	1.24 [1.09, 1.40]	9.89E-04	0.0887
rs78597169	8:117841774	rs10108200	T	C	0.196	3441	0.81 [0.71, 0.92]	9.91E-04	0.0883
rs78597169	8:117825404	rs188539646	A	G	0.016	2699	2.23 [1.41, 3.52]	5.68E-04	0.0202
rs2297038	10:30663464	rs2297038	T	C	0.434	2843	1.33 [1.18, 1.50]	2.95E-06	0.9945
rs9600669	13:76758048	rs2175075	A	G	0.090	3375	0.64 [0.54, 0.77]	2.00E-06	0.0487
rs9600669	13:76776621	rs9600669	A	G	0.086	3375	1.57 [1.30, 1.88]	1.94E-06	0.0470
rs9600669	13:76773963	rs2328973	C	G	0.086	3375	0.64 [0.53, 0.77]	1.95E-06	0.0467
rs9600669	13:76766603	rs6562946	T	C	0.090	3375	1.55 [1.29, 1.86]	2.19E-06	0.0442
rs9600669	13:76773368	rs7983570	C	G	0.090	3375	0.65 [0.54, 0.77]	2.24E-06	0.0438

rs9600669	13:76759200	rs56411210	C	G	0.086	3375	0.64 [0.53, 0.77]	2.46E-06	0.0396
rs9600669	13:76760147	rs10507848	A	G	0.086	3375	1.56 [1.29, 1.87]	2.48E-06	0.0394
rs9600669	13:76781463	rs9318413	A	T	0.091	3375	0.64 [0.54, 0.77]	2.55E-06	0.0391
rs9600669	13:76762700	rs10507849	T	C	0.086	3375	0.64 [0.53, 0.77]	2.52E-06	0.0386
rs9600669	13:76753631	rs12428519	T	G	0.090	3375	0.65 [0.54, 0.78]	2.73E-06	0.0380
rs9600669	13:76756563	rs55918529	T	C	0.090	3375	0.65 [0.54, 0.78]	2.71E-06	0.0376
rs9600669	13:76755497	rs73226165	T	G	0.090	3375	0.65 [0.54, 0.78]	2.73E-06	0.0373
rs9600669	13:76757485	rs12427641	A	G	0.090	3375	1.54 [1.29, 1.85]	2.75E-06	0.0373
rs9600669	13:76748017	rs2136340	A	G	0.087	3375	1.55 [1.29, 1.87]	2.60E-06	0.0372
rs9600669	13:76749009	rs73226155	T	C	0.090	3375	1.54 [1.29, 1.85]	2.81E-06	0.0368
rs9600669	13:76749117	rs73226156	T	C	0.090	3375	1.54 [1.29, 1.85]	2.87E-06	0.0361
rs9600669	13:76747030	rs2036717	T	C	0.090	3375	1.54 [1.29, 1.85]	2.91E-06	0.0356
rs9600669	13:76748063	rs2136339	T	C	0.090	3375	0.65 [0.54, 0.78]	2.92E-06	0.0356
rs9600669	13:76752411	rs73226162	T	C	0.090	3375	1.54 [1.28, 1.84]	2.97E-06	0.0354
rs9600669	13:76746556	rs11618016	T	G	0.090	3375	1.54 [1.28, 1.85]	2.98E-06	0.0352
rs9600669	13:76746743	rs2036718	T	C	0.090	3375	1.54 [1.28, 1.85]	2.96E-06	0.0352
rs9600669	13:76744456	rs59938144	A	C	0.090	3375	1.54 [1.28, 1.85]	3.01E-06	0.0350
rs9600669	13:76761187	rs4142522	A	G	0.085	3375	1.56 [1.29, 1.87]	3.07E-06	0.0323
rs9600669	13:76753228	rs12430486	A	G	0.090	3375	1.53 [1.28, 1.83]	4.12E-06	0.0272
rs9600669	13:76760227	rs4142523	T	C	0.088	3375	0.65 [0.54, 0.78]	4.14E-06	0.0267
rs9600669	13:76741023	rs534739393	CA	C	0.101	2843	0.67 [0.56, 0.81]	2.38E-05	0.0067
rs8003262	14:102050501	rs8003262	A	G	0.059	3023	1.73 [1.37, 2.19]	4.42E-06	0.2693
rs8003262	14:102049001	rs58241177	A	C	0.059	3023	0.58 [0.46, 0.73]	4.51E-06	0.2640
rs8003262	14:102046489	rs11849688	T	C	0.059	3023	0.59 [0.46, 0.74]	7.68E-06	0.1817
rs8003262	14:102035570	rs7158280	T	C	0.062	3023	1.60 [1.27, 2.00]	5.50E-05	0.0491
rs8003262	14:102034054	rs10134976	T	C	0.062	3023	1.60 [1.27, 2.01]	5.47E-05	0.0489
rs8003262	14:102033210	rs9324041	A	G	0.062	3023	0.63 [0.50, 0.79]	6.11E-05	0.0451
rs8003262	14:102033207	rs9324040	C	G	0.061	3023	0.63 [0.50, 0.79]	6.65E-05	0.0422
rs8003262	14:102030527	rs1997907	C	G	0.059	3023	0.62 [0.49, 0.78]	6.22E-05	0.0415
rs8003262	14:102048013	rs33927302	A	AT	0.060	2699	1.66 [1.30, 2.12]	5.56E-05	0.0402
rs149318872	15:101162067	rs149318872	G	GGA	0.049	2699	0.50 [0.37, 0.67]	3.42E-06	0.5238
rs149318872	15:101162066	rs150515319	T	TG	0.094	2699	0.67 [0.55, 0.81]	3.24E-05	0.3994
rs149318872	15:101159421	rs60819954	T	C	0.094	3265	0.73 [0.61, 0.88]	6.09E-04	0.0462
rs10421326	19:473572	rs10421326	C	G	0.174	2699	1.53 [1.31, 1.78]	5.74E-08	0.8160
rs10421326	19:469979	rs34770712	A	AG	0.189	2765	1.39 [1.21, 1.60]	3.85E-06	0.0273
rs10421326	19:470373	19:470373:A:AT	A	AT	0.167	2699	0.70 [0.60, 0.82]	4.08E-06	0.0235
rs10421326	19:465427	rs140502943	G	GTGAA	0.190	2765	1.37 [1.20, 1.57]	5.21E-06	0.0221

rs10421326	19:465302	rs10406389	A	G	0.187	2699	0.73 [0.64, 0.84]	6.56E-06	0.0176
rs10421326	19:463326	rs1968130	A	G	0.183	2909	1.36 [1.19, 1.56]	9.35E-06	0.0134
rs10421326	19:465071	rs10405754	A	G	0.187	2843	0.74 [0.65, 0.85]	1.12E-05	0.0113
rs10421326	19:466793	rs10414788	A	G	0.186	2843	0.74 [0.64, 0.84]	1.16E-05	0.0110
rs10421326	19:465889	rs28972633	T	C	0.216	2699	0.75 [0.66, 0.85]	1.33E-05	0.0101
rs827937	20:46793101	rs827937	A	T	0.248	2843	1.37 [1.21, 1.55]	1.08E-06	0.2062
rs827937	20:46792921	rs2605205	A	G	0.247	2843	1.37 [1.20, 1.55]	1.29E-06	0.1761
rs827937	20:46788676	rs228194	A	G	0.245	3441	1.33 [1.18, 1.50]	2.33E-06	0.1093
rs827937	20:46787825	rs827944	T	C	0.244	3441	0.75 [0.67, 0.85]	2.46E-06	0.1033
rs827937	20:46787378	rs827947	C	G	0.244	3441	1.33 [1.18, 1.49]	2.80E-06	0.0941
rs827937	20:46787459	rs827946	T	G	0.245	3441	0.76 [0.67, 0.85]	3.11E-06	0.0863
rs827937	20:46792944	rs827939	T	C	0.245	3375	0.76 [0.67, 0.85]	4.61E-06	0.0596
rs827937	20:46792236	rs827941	T	C	0.245	3441	0.76 [0.67, 0.85]	4.78E-06	0.0586
rs827937	20:46793073	rs67232918	A	ACACT	0.250	2699	0.74 [0.65, 0.84]	5.30E-06	0.0500
rs827937	20:46789064	20:46789064:A:AAAACAAACAAAC	A	AAAACAAACAAAC	0.188	2699	1.38 [1.20, 1.59]	8.63E-06	0.0299
**(B) Long-term varenicline effectiveness (LVE)**									
rs895545	2:121721467	rs895545	T	C	0.057	3513	0.59 [0.48, 0.73]	1.21E-06	0.0487
rs895545	2:121720125	rs4848664	T	C	0.057	3513	1.69 [1.36, 2.09]	1.64E-06	0.0388
rs895545	2:121721845	rs895546	A	T	0.056	3687	0.60 [0.48, 0.74]	1.83E-06	0.0364
rs895545	2:121723791	rs11122838	T	C	0.057	3687	0.60 [0.49, 0.74]	2.07E-06	0.0350
rs895545	2:121719268	rs13432147	T	C	0.057	3687	1.66 [1.34, 2.05]	2.50E-06	0.0299
rs895545	2:121725452	rs2677527	A	G	0.057	3687	0.61 [0.49, 0.75]	2.79E-06	0.0281
rs895545	2:121725534	rs2592596	T	C	0.057	3687	0.61 [0.49, 0.75]	2.78E-06	0.0281
rs895545	2:121726927	rs2677509	T	C	0.057	3687	0.61 [0.49, 0.75]	2.79E-06	0.0281
rs895545	2:121725418	rs2677528	A	T	0.057	3687	1.65 [1.34, 2.03]	2.80E-06	0.0280
rs895545	2:121719538	rs2166562	A	G	0.060	3336	0.60 [0.49, 0.74]	2.71E-06	0.0274
rs895545	2:121719537	rs2166563	A	C	0.060	3336	1.66 [1.34, 2.06]	2.79E-06	0.0268
rs895545	2:121718926	rs4422162	A	G	0.056	3687	1.66 [1.34, 2.06]	2.94E-06	0.0255
rs895545	2:121729323	rs2677507	A	G	0.057	3687	1.64 [1.33, 2.03]	3.68E-06	0.0224
rs895545	2:121719089	rs4625910	A	G	0.057	3687	0.61 [0.50, 0.75]	3.93E-06	0.0219
rs895545	2:121720245	rs4848667	T	C	0.057	3687	1.64 [1.33, 2.02]	4.03E-06	0.0212
rs895545	2:121718978	rs4287773	T	C	0.057	3687	1.64 [1.33, 2.02]	4.11E-06	0.0209
rs895545	2:121719171	rs4625911	A	G	0.057	3687	0.61 [0.50, 0.75]	4.12E-06	0.0208
rs895545	2:121719195	rs4625912	A	G	0.057	3687	0.61 [0.50, 0.75]	4.11E-06	0.0208
rs895545	2:121719948	rs2121435	T	C	0.057	3687	1.64 [1.33, 2.02]	4.12E-06	0.0208
rs895545	2:121720126	rs4848665	A	G	0.057	3687	0.61 [0.50, 0.75]	4.12E-06	0.0208
rs895545	2:121720191	rs4848666	T	C	0.057	3687	0.61 [0.50, 0.75]	4.12E-06	0.0208

rs895545	2:121720300	rs4848668	T	C	0.057	3687	0.61 [0.50, 0.75]	4.12E-06	0.0208
rs895545	2:121720392	rs4848669	T	C	0.057	3687	1.64 [1.33, 2.02]	4.12E-06	0.0208
rs895545	2:121720620	rs7349266	A	G	0.057	3687	1.64 [1.33, 2.02]	4.12E-06	0.0208
rs895545	2:121709536	rs12711535	T	C	0.057	3687	0.61 [0.49, 0.75]	4.13E-06	0.0203
rs895545	2:121710855	rs2084234	T	G	0.057	3687	1.64 [1.33, 2.02]	4.44E-06	0.0197
rs895545	2:121713030	rs12614482	A	G	0.057	3687	0.61 [0.50, 0.76]	4.70E-06	0.0188
rs895545	2:121720434	rs11680138	A	T	0.057	3687	1.63 [1.32, 2.01]	4.71E-06	0.0188
rs895545	2:121713203	rs13010895	A	G	0.057	3687	0.61 [0.50, 0.76]	4.71E-06	0.0188
rs895545	2:121729172	rs2243855	T	C	0.057	3687	0.61 [0.50, 0.76]	4.85E-06	0.0185
rs895545	2:121709786	rs12711536	T	C	0.057	3687	0.61 [0.50, 0.76]	4.86E-06	0.0184
rs895545	2:121716605	rs753900	C	G	0.057	3687	1.63 [1.32, 2.02]	4.87E-06	0.0183
rs895545	2:121712361	rs4848660	T	C	0.057	3687	1.63 [1.32, 2.01]	4.92E-06	0.0181
rs895545	2:121712381	rs4848661	T	C	0.057	3687	0.61 [0.50, 0.76]	4.92E-06	0.0181
rs895545	2:121712418	rs4848662	A	G	0.057	3687	0.61 [0.50, 0.76]	4.91E-06	0.0181
rs895545	2:121712441	rs4848663	T	C	0.057	3687	1.63 [1.32, 2.01]	4.92E-06	0.0181
rs895545	2:121712507	rs4848134	A	C	0.057	3687	1.63 [1.32, 2.01]	4.92E-06	0.0181
rs895545	2:121711860	rs11122837	A	G	0.057	3687	0.61 [0.50, 0.76]	4.94E-06	0.0181
rs895545	2:121712260	rs11689760	A	C	0.057	3687	1.63 [1.32, 2.01]	4.94E-06	0.0181
rs895545	2:121713041	rs12617878	T	C	0.057	3687	1.63 [1.32, 2.01]	5.19E-06	0.0176
rs895545	2:121719392	rs7420788	A	G	0.058	3687	1.59 [1.29, 1.95]	1.19E-05	0.0098
rs895545	2:121692477	rs2166565	A	G	0.052	3687	1.58 [1.27, 1.97]	4.53E-05	0.0032
rs895545	2:121695699	rs895550	A	G	0.054	3687	0.64 [0.52, 0.79]	4.98E-05	0.0031
rs895545	2:121708159	rs11122835	T	G	0.053	3687	1.57 [1.26, 1.96]	4.97E-05	0.0030
rs895545	2:121709091	rs11681136	A	G	0.053	3687	0.64 [0.51, 0.79]	5.03E-05	0.0030
rs7712227	5:118656769	rs7712227	A	G	0.060	3687	0.59 [0.47, 0.73]	2.56E-06	0.0317
rs7712227	5:118671118	rs7725244	A	G	0.062	3886	0.61 [0.50, 0.75]	4.33E-06	0.0248
rs7712227	5:118666751	rs72786143	T	C	0.062	3886	1.63 [1.32, 2.02]	4.80E-06	0.0231
rs7712227	5:118670540	rs72786161	T	G	0.062	3886	1.63 [1.32, 2.02]	4.75E-06	0.0230
rs7712227	5:118659359	rs72786136	A	G	0.061	3886	0.62 [0.50, 0.76]	7.42E-06	0.0167
rs7712227	5:118675017	rs7737934	T	C	0.063	3886	1.60 [1.30, 1.97]	8.09E-06	0.0162
rs7712227	5:118660784	rs66535134	A	T	0.062	3687	1.62 [1.31, 2.01]	7.88E-06	0.0156
rs7712227	5:118675446	rs3813305	T	G	0.050	3513	1.76 [1.38, 2.24]	4.89E-06	0.0145
rs7712227	5:118667642	rs7704398	T	C	0.063	3886	0.63 [0.51, 0.77]	1.04E-05	0.0136
rs7712227	5:118674701	rs7724178	T	C	0.063	3886	1.59 [1.30, 1.96]	1.06E-05	0.0133
rs7712227	5:118674893	rs7704334	A	G	0.063	3886	1.59 [1.30, 1.96]	1.06E-05	0.0133
rs7712227	5:118674923	rs7704487	T	C	0.063	3886	0.63 [0.51, 0.77]	1.06E-05	0.0133
rs7712227	5:118674774	rs7724204	T	C	0.063	3886	1.59 [1.30, 1.96]	1.06E-05	0.0133

rs7712227	5:118674623	rs7723976	T	C	0.063	3886	1.59 [1.30, 1.96]	1.07E-05	0.0133
rs7712227	5:118674652	rs7704291	A	G	0.063	3886	0.63 [0.51, 0.77]	1.06E-05	0.0133
rs7712227	5:118674284	rs6862831	C	G	0.063	3886	0.63 [0.51, 0.77]	1.12E-05	0.0129
rs7712227	5:118674290	rs6862517	C	G	0.063	3886	1.59 [1.29, 1.96]	1.12E-05	0.0128
rs7712227	5:118673697	rs17164572	T	C	0.063	3886	0.63 [0.51, 0.77]	1.14E-05	0.0126
rs7712227	5:118672441	rs17145198	A	G	0.063	3886	1.59 [1.29, 1.96]	1.15E-05	0.0125
rs7712227	5:118672472	rs11955852	A	C	0.063	3886	1.59 [1.29, 1.96]	1.15E-05	0.0125
rs7712227	5:118661434	rs111332768	T	C	0.064	3886	1.58 [1.29, 1.94]	1.19E-05	0.0125
rs7712227	5:118661649	rs67311526	T	C	0.064	3886	1.58 [1.29, 1.95]	1.18E-05	0.0124
rs7712227	5:118674055	rs6862320	A	G	0.063	3886	0.63 [0.51, 0.77]	1.16E-05	0.0124
rs7712227	5:118674747	rs757741772	CA	C	0.061	3162	0.58 [0.46, 0.74]	6.96E-06	0.0123
rs7712227	5:118669224	rs11959659	A	G	0.063	3886	0.63 [0.51, 0.77]	1.19E-05	0.0123
rs7712227	5:118671834	rs60507834	A	G	0.063	3886	1.59 [1.29, 1.95]	1.20E-05	0.0123
rs7712227	5:118672058	rs17145194	A	G	0.063	3886	1.59 [1.29, 1.95]	1.20E-05	0.0123
rs7712227	5:118672137	rs17145196	A	C	0.063	3886	0.63 [0.51, 0.77]	1.20E-05	0.0123
rs7712227	5:118674041	rs6883394	T	C	0.063	3886	1.59 [1.29, 1.96]	1.18E-05	0.0122
rs7712227	5:118669780	rs72786157	A	G	0.063	3886	1.59 [1.29, 1.95]	1.21E-05	0.0122
rs7712227	5:118671110	rs7707221	T	C	0.063	3886	1.59 [1.29, 1.95]	1.21E-05	0.0122
rs7712227	5:118668406	rs6868702	A	G	0.063	3886	1.59 [1.29, 1.95]	1.21E-05	0.0121
rs7712227	5:118668538	rs6869017	C	G	0.063	3886	1.59 [1.29, 1.95]	1.21E-05	0.0121
rs7712227	5:118672197	rs73790807	A	G	0.063	3886	1.59 [1.29, 1.95]	1.21E-05	0.0121
rs7712227	5:118668381	rs6868688	A	C	0.063	3886	1.59 [1.29, 1.95]	1.21E-05	0.0121
rs7712227	5:118670591	rs17145189	A	G	0.063	3886	0.63 [0.51, 0.77]	1.21E-05	0.0121
rs7712227	5:118671062	rs11951771	T	G	0.063	3886	1.59 [1.29, 1.95]	1.21E-05	0.0121
rs7712227	5:118667909	rs72786149	T	G	0.063	3886	1.59 [1.29, 1.95]	1.21E-05	0.0121
rs7712227	5:118667979	rs72786152	C	G	0.063	3886	1.59 [1.29, 1.95]	1.21E-05	0.0121
rs7712227	5:118671462	rs7707736	T	C	0.063	3886	1.59 [1.29, 1.95]	1.20E-05	0.0120
rs7712227	5:118671517	rs7725878	A	G	0.063	3886	0.63 [0.51, 0.77]	1.20E-05	0.0120
rs7712227	5:118667610	rs7704386	C	G	0.063	3886	1.59 [1.29, 1.95]	1.21E-05	0.0120
rs7712227	5:118667072	rs72786144	A	T	0.063	3886	0.63 [0.51, 0.78]	1.22E-05	0.0120
rs7712227	5:118667211	rs7703998	A	G	0.063	3886	0.63 [0.51, 0.78]	1.22E-05	0.0120
rs7712227	5:118666627	rs72786142	A	C	0.063	3886	0.63 [0.51, 0.78]	1.22E-05	0.0119
rs7712227	5:118663397	rs7711312	A	G	0.063	3886	0.63 [0.51, 0.78]	1.23E-05	0.0119
rs7712227	5:118674020	rs6862299	A	G	0.063	3886	0.63 [0.51, 0.77]	1.23E-05	0.0119
rs7712227	5:118667253	rs7704026	A	G	0.063	3886	0.63 [0.51, 0.78]	1.24E-05	0.0119
rs7712227	5:118674026	rs6861849	A	G	0.063	3886	1.59 [1.29, 1.96]	1.25E-05	0.0118
rs7712227	5:118663310	rs11958957	T	C	0.063	3886	0.63 [0.51, 0.78]	1.25E-05	0.0118

rs7712227	5:118663373	rs7710939	T	C	0.063	3886	0.63 [0.51, 0.78]	1.25E-05	0.0118
rs7712227	5:118674429	rs7703577	A	C	0.063	3886	1.59 [1.29, 1.95]	1.26E-05	0.0115
rs7712227	5:118675246	rs3813301	A	G	0.063	3886	1.58 [1.29, 1.95]	1.31E-05	0.0113
rs7712227	5:118661206	rs181617086	A	G	0.063	3886	0.64 [0.52, 0.78]	1.79E-05	0.0091
rs7712227	5:118667451	rs7724082	T	C	0.064	3886	1.57 [1.28, 1.92]	1.87E-05	0.0089
rs7712227	5:118671091	rs11954854	A	C	0.064	3886	1.57 [1.28, 1.93]	1.88E-05	0.0088
rs7712227	5:118660500	rs11956285	T	G	0.063	3886	0.64 [0.52, 0.78]	1.87E-05	0.0087
rs7712227	5:118660569	rs11959729	T	C	0.063	3886	1.57 [1.28, 1.93]	1.87E-05	0.0087
rs7712227	5:118659537	rs60161062	A	G	0.063	3886	0.64 [0.52, 0.78]	1.87E-05	0.0087
rs7712227	5:119246341	rs34865044	A	AT	0.247	3535	1.27 [1.13, 1.43]	4.60E-05	0.0084
rs7712227	5:118660289	rs11956215	A	G	0.063	3886	0.64 [0.52, 0.78]	2.01E-05	0.0082
rs7712227	5:118658731	rs7722699	A	G	0.062	3886	0.64 [0.52, 0.78]	2.04E-05	0.0081
rs7712227	5:118658702	rs7722687	T	G	0.063	3886	0.64 [0.52, 0.78]	2.09E-05	0.0080
rs7712227	5:118637787	rs72786120	A	G	0.039	3513	0.53 [0.40, 0.70]	9.01E-06	0.0060
rs7712227	5:118659296	rs60756572	C	G	0.063	3886	1.55 [1.26, 1.90]	3.25E-05	0.0058
rs7712227	5:118628651	rs60599743	A	T	0.054	3687	0.61 [0.48, 0.77]	2.53E-05	0.0053
rs7712227	5:118672213	5:118672213:G:GACTTCTTAT	G	GACTTCTTAT	0.064	3535	0.63 [0.51, 0.79]	3.65E-05	0.0048
rs7712227	5:118675405	rs3813304	A	G	0.051	3712	1.63 [1.29, 2.06]	3.66E-05	0.0040
rs7712227	5:118661697	rs11960654	T	C	0.066	3886	1.51 [1.23, 1.84]	6.06E-05	0.0039
rs7712227	5:118665726	rs67154545	A	G	0.066	3886	1.51 [1.23, 1.85]	6.30E-05	0.0037
rs7712227	5:118665801	rs11956042	C	G	0.066	3886	1.51 [1.23, 1.85]	6.30E-05	0.0037
rs7712227	5:118666249	rs11960246	T	C	0.066	3886	1.51 [1.23, 1.85]	6.30E-05	0.0037
rs7712227	5:118666603	rs72786141	A	G	0.066	3886	1.51 [1.23, 1.85]	6.26E-05	0.0037
rs7712227	5:118665520	rs11959451	T	G	0.066	3886	1.51 [1.23, 1.85]	6.31E-05	0.0037
rs7712227	5:118665158	rs4392658	T	C	0.066	3886	1.51 [1.23, 1.85]	6.34E-05	0.0037
rs7712227	5:118665421	rs1876737	C	G	0.066	3886	0.66 [0.54, 0.81]	6.32E-05	0.0037
rs7712227	5:118663542	rs7711101	A	C	0.066	3886	1.51 [1.23, 1.85]	6.33E-05	0.0037
rs7712227	5:118663979	rs6875361	A	T	0.066	3886	1.51 [1.23, 1.85]	6.34E-05	0.0037
rs7712227	5:118664573	rs11950366	T	C	0.066	3886	1.51 [1.23, 1.85]	6.34E-05	0.0037
rs7712227	5:118664645	rs11959964	T	C	0.066	3886	0.66 [0.54, 0.81]	6.34E-05	0.0037
rs7712227	5:118665062	rs67708265	A	G	0.066	3886	1.51 [1.23, 1.85]	6.34E-05	0.0037
rs7712227	5:118670361	rs72786159	T	C	0.064	3535	1.56 [1.26, 1.93]	5.34E-05	0.0037
rs7712227	5:118672208	rs111815603	C	G	0.064	3535	1.56 [1.26, 1.93]	5.34E-05	0.0037
rs7712227	5:118670187	5:118670187:A:AC	A	AC	0.064	3535	0.64 [0.52, 0.80]	5.34E-05	0.0037
rs7712227	5:118627151	rs73794110	T	C	0.053	3886	0.63 [0.50, 0.79]	5.44E-05	0.0033
rs7712227	5:118649135	rs7701688	A	T	0.052	3687	1.62 [1.28, 2.04]	5.02E-05	0.0033
rs7712227	5:118616559	rs67186626	T	C	0.053	3886	0.63 [0.50, 0.79]	5.91E-05	0.0031

rs7712227	5:118621929	rs73794107	A	G	0.053	3886	1.58 [1.27, 1.98]	5.99E-05	0.0031
rs7712227	5:118620885	rs72786113	T	C	0.053	3886	1.58 [1.27, 1.98]	6.05E-05	0.0031
rs7712227	5:118628798	rs72786118	A	G	0.053	3886	1.58 [1.26, 1.98]	6.28E-05	0.0030
rs7712227	5:118661203	rs188811851	T	C	0.066	3886	1.50 [1.22, 1.83]	8.78E-05	0.0029
rs7712227	5:119291969	rs34618848	G	GA	0.388	3535	1.22 [1.10, 1.34]	1.64E-04	0.0028
rs7712227	5:118619536	rs10519576	A	G	0.053	3886	0.63 [0.51, 0.79]	6.78E-05	0.0028
rs7712227	5:118647014	rs58131651	T	C	0.053	3886	0.63 [0.50, 0.79]	6.75E-05	0.0028
rs7712227	5:118635627	rs6892643	C	G	0.053	3886	1.58 [1.26, 1.97]	6.93E-05	0.0028
rs7712227	5:118635662	rs6892980	A	G	0.053	3886	0.63 [0.51, 0.79]	6.93E-05	0.0028
rs7712227	5:118640218	rs6879962	A	T	0.053	3886	1.58 [1.26, 1.97]	7.01E-05	0.0028
rs7712227	5:118637058	rs56319623	A	G	0.053	3886	0.63 [0.51, 0.79]	7.00E-05	0.0028
rs7712227	5:118643238	rs72786122	A	G	0.053	3886	0.63 [0.50, 0.79]	6.89E-05	0.0028
rs7712227	5:118664034	rs11421168	CA	C	0.066	3162	0.64 [0.51, 0.80]	7.45E-05	0.0028
rs7712227	5:118639499	rs10519581	A	G	0.053	3886	1.58 [1.26, 1.97]	7.03E-05	0.0028
rs7712227	5:118646065	rs72786125	T	C	0.053	3886	0.63 [0.50, 0.79]	6.93E-05	0.0028
rs7712227	5:118645320	rs17145135	A	G	0.053	3886	0.63 [0.51, 0.79]	7.14E-05	0.0027
rs7712227	5:118626215	rs72786116	A	G	0.053	3886	0.63 [0.50, 0.79]	7.03E-05	0.0027
rs7712227	5:118677967	rs67967956	C	G	0.061	3886	1.52 [1.23, 1.88]	9.58E-05	0.0025
rs78695099	6:99997165	rs2894870	A	T	0.036	3162	0.47 [0.35, 0.63]	2.84E-07	0.3621
rs78695099	6:99997166	rs2894871	A	C	0.035	3162	0.48 [0.36, 0.64]	5.09E-07	0.2473
rs78695099	6:100195347	rs78695099	A	G	0.026	3162	0.42 [0.30, 0.58]	2.05E-07	0.2030
rs78695099	6:100022491	rs75713647	T	C	0.025	3162	2.21 [1.60, 3.07]	1.81E-06	0.0623
rs78695099	6:100271658	rs540690087	A	G	0.074	3162	1.50 [1.23, 1.83]	5.45E-05	0.0349
rs78695099	6:99901767	rs148375451	T	C	0.026	3513	2.02 [1.48, 2.76]	9.90E-06	0.0285
rs78695099	6:100200753	rs74401013	T	C	0.088	3687	1.38 [1.17, 1.63]	1.42E-04	0.0211
rs35443123	7:85173800	rs35443123	A	AT	0.434	3535	0.79 [0.72, 0.88]	4.86E-06	0.6095
rs35443123	7:85142693	rs142645420	CCTAT	C	0.421	3361	0.81 [0.73, 0.90]	6.04E-05	0.0640
rs35443123	7:85157517	rs9942699	A	C	0.388	4131	1.21 [1.10, 1.33]	7.17E-05	0.0536
rs35443123	7:85157662	rs6953439	A	G	0.423	4131	1.20 [1.09, 1.32]	1.47E-04	0.0285
rs35443123	7:85166174	rs35499102	A	G	0.423	4131	0.84 [0.76, 0.92]	1.59E-04	0.0263
rs35443123	7:85160755	rs6951823	T	G	0.423	4131	1.20 [1.09, 1.31]	1.66E-04	0.0259
rs35443123	7:85174307	rs28432363	A	G	0.431	4131	0.84 [0.76, 0.92]	1.73E-04	0.0249
rs35443123	7:85168495	rs13230012	T	C	0.314	3535	0.82 [0.74, 0.91]	2.18E-04	0.0204
rs35443123	7:85004755	rs77649879	T	C	0.235	4093	1.23 [1.10, 1.37]	2.63E-04	0.0174
rs35443123	7:85177938	rs10252600	T	C	0.430	4131	1.19 [1.08, 1.30]	3.56E-04	0.0130
rs35443123	7:85116129	rs12704136	T	C	0.426	4131	0.84 [0.77, 0.93]	4.34E-04	0.0108
rs35443123	7:85113311	rs1554847	A	G	0.427	4131	1.18 [1.08, 1.30]	4.75E-04	0.0102

rs35443123	7:85123452	rs35923742	A	T	0.426	4131	1.18 [1.08, 1.30]	5.24E-04	0.0094
rs35443123	7:85112099	rs55662263	T	C	0.427	4131	0.85 [0.77, 0.93]	5.53E-04	0.0089
rs35443123	7:85110875	rs55804990	A	G	0.427	4131	1.18 [1.07, 1.30]	5.65E-04	0.0088
rs35443123	7:85120974	rs34596135	T	C	0.426	3932	1.18 [1.08, 1.30]	5.71E-04	0.0087
rs35443123	7:85121064	rs10499889	T	C	0.427	4131	1.18 [1.07, 1.29]	6.11E-04	0.0082
rs35443123	7:85155661	rs2140458	C	G	0.323	4131	1.19 [1.07, 1.31]	6.47E-04	0.0077
rs766169	22:47680100	rs766169	T	G	0.051	3513	1.84 [1.47, 2.29]	6.68E-08	0.6043
rs766169	22:47679901	rs766168	T	C	0.050	3513	1.79 [1.43, 2.23]	2.70E-07	0.2100
rs766169	22:47679506	rs58656950	A	G	0.049	3513	1.80 [1.44, 2.25]	3.12E-07	0.1809

Abbreviations: MAF, minor allele frequency; N, sample size; OR, odds ratio; PIP, posterior inclusion probability.

Supplementary Table 5 Look-up of variants identified in association studies of varenicline-related phenotypes and smoking cessation in SVE and LVE Stage 1 GWASs.

Reference	Variant	Chr:Position (hg19)	Gene	EA	Stage 1 GWAS of SVE			Stage 1 GWAS of LVE		
					EAF	OR [95% CI]	*p*-value	EAF	OR [95% CI]	*p*-value
**Varenicline efficacy (from candidate gene studies)**										
9	rs4292956	1:154548946	*CHRNB2*	T	0.075	0.97 [0.79, 1.18]	7.59×10^{-1}	0.073	1.03 [0.86, 1.24]	7.19×10^{-1}
9	rs3811450	1:154551032	*CHRNB2*	T	0.076	0.96 [0.79, 1.17]	6.73×10^{-1}	0.074	1.01 [0.84, 1.21]	9.42×10^{-1}
9	rs6494212	15:32385119	*CHRNA7*	T	0.317	0.93 [0.83, 1.04]	2.10×10^{-1}	0.314	1.05 [0.95, 1.17]	3.59×10^{-1}
9	rs2938674	15:78757913	*IREB2*	A	0.205	1.00 [0.88, 1.13]	9.95×10^{-1}	0.206	1.07 [0.96, 1.20]	2.08×10^{-1}
9	rs7164594	15:78803057	*HYKK*	T	0.205	1.04 [0.92, 1.18]	5.09×10^{-1}	0.204	1.10 [0.98, 1.24]	9.16×10^{-2}
10	rs16969968	15:78882925	*CHRNA5*	A	0.351	0.94 [0.84, 1.04]	2.29×10^{-1}	0.350	0.92 [0.83, 1.01]	7.61×10^{-2}
9	rs518425	15:78883813	*CHRNA5*	G	0.272	1.15 [0.92, 1.15]	6.12×10^{-1}	0.271	1.07 [0.97, 1.19]	1.71×10^{-1}
11	rs8109525	19:41491918	*CYP2B6*	G	0.163	1.11 [0.90, 1.11]	9.71×10^{-1}	0.162	1.01 [0.92, 1.12]	7.76×10^{-1}
9	rs2236196	20:61977556	*CHRNA4*	G	0.339	1.07 [0.85, 1.07]	4.31×10^{-1}	0.340	0.86 [0.77, 0.96]	5.29×10^{-3}
9	rs3787138	20:61979224	*CHRNA4*	G	0.253	1.09 [0.80, 1.09]	3.83×10^{-1}	0.252	0.83 [0.71, 0.95]	9.18×10^{-3}
9	rs6062899	20:61979793	*CHRNA4*	G	0.124	1.11 [0.86, 1.11]	7.31×10^{-1}	0.121	0.85 [0.75, 0.96]	1.09×10^{-2}
12	rs1044396	20:61981134	*CHRNA4*	T	0.175	1.13 [0.92, 1.13]	6.69×10^{-1}	0.173	0.96 [0.88, 1.06]	4.16×10^{-1}
**Nausea severity post-varenicline treatment**										
13	rs2072660	1:154548721	*CHRNB2*	T	0.241	1.04 [0.92, 1.16]	5.58×10^{-1}	0.238	1.04 [0.93, 1.15]	5.15×10^{-1}
13	rs2072661	1:154548880	*CHRNB2*	G	0.756	0.96 [0.85, 1.08]	4.67×10^{-1}	0.758	0.96 [0.87, 1.07]	4.84×10^{-1}
13	rs4292956	1:154548946	*CHRNB2*	C	0.925	1.03 [0.84, 1.26]	7.59×10^{-1}	0.927	0.97 [0.80, 1.16]	7.19×10^{-1}
13	rs2302764	17:7360110	*CHRNB1*	C	0.163	0.94 [0.82, 1.08]	3.77×10^{-1}	0.162	1.02 [0.89, 1.15]	8.05×10^{-1}
**Smoking cessation**										
14	rs7521775	1:22475649	Intergenic	C	0.590	1.12 [1.01, 1.24]	2.69×10^{-2}	0.587	1.06 [0.97, 1.16]	2.27×10^{-1}
14	rs10794514	1:28659696	*MED18*	T	0.427	1.00 [0.91, 1.11]	9.63×10^{-1}	0.427	1.00 [0.91, 1.10]	9.85×10^{-1}
14	rs3791151	1:44078602	*PTPRF*	C	0.662	0.99 [0.89, 1.10]	8.34×10^{-1}	0.665	0.97 [0.88, 1.07]	4.96×10^{-1}
14	rs1171279	1:65988493	*LEPR*	T	0.271	1.03 [0.92, 1.15]	6.32×10^{-1}	0.265	1.00 [0.90, 1.11]	9.65×10^{-1}
14	rs6677439	1:73837907	Intergenic	C	0.548	1.02 [0.91, 1.14]	7.48×10^{-1}	0.549	1.02 [0.92, 1.14]	6.80×10^{-1}
14	rs699536	1:90934652	Intergenic	G	0.569	1.01 [0.92, 1.12]	8.09×10^{-1}	0.575	0.99 [0.91, 1.09]	9.09×10^{-1}
14	rs17379561	1:98340139	*DPYD*	T	0.144	1.02 [0.88, 1.18]	7.93×10^{-1}	0.148	1.10 [0.97, 1.25]	1.53×10^{-1}

[14]	rs12727810	1:162068428	*NOS1AP*	G	0.371	1.01 [0.91, 1.13]	7.87×10^{-1}	0.371	1.01 [0.92, 1.11]	8.39×10^{-1}
[14]	rs9724968	1:162069609	*NOS1AP*	T	0.374	1.00 [0.90, 1.12]	9.88×10^{-1}	0.376	1.01 [0.91, 1.12]	8.70×10^{-1}
[14]	rs6673326	1:169420973	*CCDC181*	G	0.375	1.09 [0.98, 1.22]	1.20×10^{-1}	0.373	1.00 [0.90, 1.11]	9.99×10^{-1}
[14]	rs6425284	1:174499977	*RABGAP1L*	G	0.707	1.11 [0.98, 1.27]	1.12×10^{-1}	0.706	1.03 [0.91, 1.17]	6.09×10^{-1}
[14]	rs12046747	1:204593696	*LRRN2*	G	0.792	0.93 [0.82, 1.05]	2.19×10^{-1}	0.792	1.05 [0.93, 1.17]	4.47×10^{-1}
[14]	rs72781639	2:24204148	*UBXN2A*	G	0.156	1.01 [0.87, 1.16]	9.30×10^{-1}	0.154	1.04 [0.91, 1.18]	5.94×10^{-1}
[14]	rs12614418	2:35730212	Intergenic	G	0.442	1.02 [0.91, 1.14]	7.34×10^{-1}	0.439	1.03 [0.93, 1.14]	5.57×10^{-1}
[14]	rs7569203	2:45154418	*LINC01833*	C	0.318	0.89 [0.80, 0.99]	3.26×10^{-2}	0.325	0.94 [0.85, 1.04]	2.34×10^{-1}
[14]	rs1369294	2:50232133	*NRXN1*	T	0.472	0.97 [0.88, 1.07]	5.98×10^{-1}	0.470	0.98 [0.90, 1.08]	7.25×10^{-1}
[14]	rs10191428	2:62725407	Intergenic	C	0.903	1.05 [0.88, 1.24]	6.12×10^{-1}	0.903	0.95 [0.81, 1.11]	5.28×10^{-1}
[14]	rs7598901	2:73675844	*ALMS1*	C	0.384	1.04 [0.94, 1.16]	4.36×10^{-1}	0.380	1.05 [0.95, 1.15]	3.35×10^{-1}
[14]	rs2916251	2:104447052	Intergenic	T	0.510	1.03 [0.92, 1.16]	5.90×10^{-1}	0.506	1.04 [0.93, 1.15]	5.04×10^{-1}
[14]	rs1374328	2:166182354	*SCN2A*	T	0.756	0.97 [0.86, 1.09]	5.90×10^{-1}	0.754	1.04 [0.94, 1.16]	4.39×10^{-1}
[14]	rs78590213	2:181920762	*UBE2E3*	T	0.707	1.05 [0.94, 1.17]	3.96×10^{-1}	0.711	1.00 [0.90, 1.11]	9.77×10^{-1}
[14]	rs56222728	2:186135147	Intergenic	T	0.610	1.06 [0.95, 1.18]	3.19×10^{-1}	0.607	1.12 [1.01, 1.24]	3.49×10^{-2}
[14]	rs12693975	2:203720745	*ICA1L*	A	0.822	1.02 [0.90, 1.17]	7.16×10^{-1}	0.826	1.02 [0.90, 1.15]	7.78×10^{-1}
[14]	rs34762726	3:49689210	*BSN*	G	0.720	0.91 [0.81, 1.02]	9.32×10^{-2}	0.723	0.94 [0.85, 1.04]	2.16×10^{-1}
[14]	rs7613360	3:49916710	Intergenic	T	0.411	0.95 [0.86, 1.05]	3.13×10^{-1}	0.414	0.95 [0.87, 1.05]	3.25×10^{-1}
[14]	rs6803651	3:64431730	Intergenic	G	0.565	0.99 [0.90, 1.10]	8.69×10^{-1}	0.566	0.93 [0.85, 1.02]	1.31×10^{-1}
[14]	rs9833290	3:77312267	*ROBO2*	C	0.402	0.98 [0.88, 1.08]	6.61×10^{-1}	0.408	0.98 [0.89, 1.08]	6.79×10^{-1}
[14]	rs2171141	3:85177049	*CADM2*	C	0.437	1.07 [0.97, 1.18]	1.86×10^{-1}	0.439	1.06 [0.97, 1.16]	2.10×10^{-1}
[14]	rs1870008	3:107954891	Intergenic	T	0.605	0.94 [0.85, 1.04]	2.36×10^{-1}	0.601	0.97 [0.89, 1.07]	5.95×10^{-1}
[14]	rs1711161	3:136080537	*STAG1*	T	0.749	1.06 [0.95, 1.19]	3.12×10^{-1}	0.749	1.08 [0.97, 1.20]	1.80×10^{-1}
[14]	rs36035502	4:35580129	Intergenic	C	0.194	0.96 [0.84, 1.10]	5.42×10^{-1}	0.196	0.84 [0.74, 0.96]	8.65×10^{-3}
[14]	rs75096215	4:35582194	Intergenic	G	0.195	0.93 [0.82, 1.05]	2.41×10^{-1}	0.198	0.86 [0.76, 0.97]	1.24×10^{-2}
[14]	rs76757555	4:35582979	Intergenic	A	0.195	0.93 [0.82, 1.05]	2.38×10^{-1}	0.198	0.86 [0.76, 0.97]	1.26×10^{-2}
[14]	rs77698860	4:35582980	Intergenic	T	0.195	0.93 [0.82, 1.05]	2.40×10^{-1}	0.198	0.86 [0.76, 0.97]	1.26×10^{-2}
[14]	rs11937919	4:35583243	Intergenic	G	0.196	0.93 [0.82, 1.05]	2.43×10^{-1}	0.199	0.86 [0.76, 0.97]	1.20×10^{-2}
[14]	rs115737563	4:42140389	*BEND4*	A	0.197	1.12 [0.98, 1.28]	8.33×10^{-2}	0.194	1.06 [0.93, 1.19]	3.80×10^{-1}
[14]	rs62310097	4:93499316	*GRID2*	T	0.362	0.99 [0.88, 1.11]	8.35×10^{-1}	0.362	0.95 [0.86, 1.06]	3.80×10^{-1}
[14]	rs11721364	4:105427978	*CXXC4-AS1*	C	0.679	1.04 [0.93, 1.15]	5.20×10^{-1}	0.675	1.00 [0.91, 1.10]	9.90×10^{-1}

[14]	rs1000350	4:113044466	Intergenic	T	0.375	0.99 [0.89, 1.10]	8.69×10^{-1}	0.378	1.01 [0.91, 1.12]	8.61×10^{-1}
[14]	rs55669488	4:140943275	*MAML3*	T	0.642	1.03 [0.93, 1.14]	6.09×10^{-1}	0.638	1.03 [0.93, 1.13]	6.05×10^{-1}
[14]	rs7668995	4:147919278	Intergenic	T	0.720	1.09 [0.97, 1.21]	1.43×10^{-1}	0.722	0.97 [0.87, 1.07]	5.36×10^{-1}
[14]	rs4691521	4:159802373	*FNIP2*	G	0.431	1.00 [0.89, 1.11]	9.45×10^{-1}	0.431	1.02 [0.92, 1.13]	6.65×10^{-1}
[14]	rs7705852	5:59855859	Intergenic	T	0.165	1.02 [0.89, 1.17]	7.35×10^{-1}	0.168	1.12 [0.99, 1.26]	8.49×10^{-2}
[14]	rs329120	5:133861756	*JADE2*	C	0.581	1.05 [0.95, 1.16]	3.24×10^{-1}	0.581	1.05 [0.96, 1.15]	2.92×10^{-1}
[14]	rs4705014	5:155852315	*SGCD*	G	0.377	1.03 [0.93, 1.14]	6.02×10^{-1}	0.374	1.08 [0.98, 1.19]	1.02×10^{-1}
[14]	rs263886	5:165170651	Intergenic	A	0.509	0.98 [0.88, 1.08]	6.34×10^{-1}	0.506	0.96 [0.88, 1.06]	4.49×10^{-1}
[14]	rs11738110	5:166992760	*TENM2*	T	0.378	0.97 [0.87, 1.07]	5.18×10^{-1}	0.373	1.04 [0.95, 1.15]	3.86×10^{-1}
[14]	rs2073526	6:26374658	*BTN3A2*	G	0.454	0.93 [0.84, 1.02]	1.32×10^{-1}	0.457	0.92 [0.84, 1.01]	8.35×10^{-2}
[14]	rs13203795	6:33583732	Intergenic	A	0.100	1.07 [0.89, 1.28]	4.81×10^{-1}	0.102	1.05 [0.88, 1.24]	5.88×10^{-1}
[14]	rs7778443	7:32314690	*PDE1C*	T	0.403	1.11 [1.01, 1.23]	3.93×10^{-2}	0.397	1.07 [0.98, 1.18]	1.39×10^{-1}
[14]	rs62459050	7:71319137	*CALN1*	G	0.360	0.89 [0.80, 0.99]	3.52×10^{-2}	0.363	0.95 [0.86, 1.05]	2.98×10^{-1}
[14]	rs113067414	7:97391962	Intergenic	G	0.758	0.98 [0.87, 1.10]	7.18×10^{-1}	0.757	0.97 [0.87, 1.09]	6.41×10^{-1}
[14]	rs13245429	7:97394886	Intergenic	G	0.795	0.96 [0.84, 1.10]	5.30×10^{-1}	0.796	0.94 [0.83, 1.07]	3.60×10^{-1}
[14]	rs7794285	7:104849737	*SRPK2*	A	0.451	0.95 [0.86, 1.05]	2.78×10^{-1}	0.456	1.00 [0.91, 1.09]	9.47×10^{-1}
[14]	rs4265129	7:113591217	Intergenic	A	0.373	1.16 [1.04, 1.28]	6.15×10^{-3}	0.372	1.02 [0.93, 1.13]	6.45×10^{-1}
[14]	rs62474673	7:114973194	Intergenic	T	0.499	0.98 [0.89, 1.08]	7.21×10^{-1}	0.499	1.01 [0.92, 1.10]	9.15×10^{-1}
[14]	rs12154872	7:117512932	*CTTNBP2*	G	0.555	0.96 [0.86, 1.06]	3.89×10^{-1}	0.560	0.97 [0.88, 1.06]	4.58×10^{-1}
[14]	rs2098112	7:153487944	*DPP6*	A	0.482	0.91 [0.83, 1.01]	7.22×10^{-2}	0.482	0.99 [0.91, 1.09]	8.73×10^{-1}
[14]	rs3807890	7:155571603	*RBM33*	G	0.198	1.01 [0.89, 1.15]	8.58×10^{-1}	0.199	0.97 [0.86, 1.10]	6.35×10^{-1}
[14]	rs11991338	8:9288464	Intergenic	A	0.178	0.91 [0.79, 1.05]	1.82×10^{-1}	0.177	0.99 [0.87, 1.13]	8.74×10^{-1}
[14]	rs2736268	8:11188532	*SLC35G5*	C	0.534	0.98 [0.88, 1.09]	6.91×10^{-1}	0.530	0.98 [0.89, 1.09]	7.65×10^{-1}
[14]	rs11773992	8:12667804	*LINC00681*|*LOC340357*	C	0.188	0.92 [0.81, 1.05]	2.18×10^{-1}	0.188	0.95 [0.84, 1.07]	3.82×10^{-1}
[14]	rs11776293	8:27418429	Intergenic	C	0.834	0.98 [0.85, 1.12]	7.40×10^{-1}	0.833	0.91 [0.80, 1.03]	1.30×10^{-1}
[14]	rs1565735	8:27426077	Intergenic	T	0.812	0.92 [0.81, 1.04]	1.91×10^{-1}	0.810	0.94 [0.83, 1.06]	2.91×10^{-1}
[14]	rs11990422	8:42582955	*CHRNB3*	G	0.897	1.00 [0.84, 1.17]	9.56×10^{-1}	0.898	1.06 [0.91, 1.24]	4.69×10^{-1}
[14]	rs10738785	9:27919960	Intergenic	A	0.350	0.96 [0.86, 1.06]	3.99×10^{-1}	0.355	0.93 [0.85, 1.03]	1.71×10^{-1}
[14]	rs10819036	9:127890913	*SCAI*	T	0.700	0.98 [0.88, 1.10]	7.52×10^{-1}	0.700	1.01 [0.91, 1.11]	8.87×10^{-1}
[14]	rs4837011	9:127923014	*PPP6C*	G	0.699	0.93 [0.82, 1.04]	1.96×10^{-1}	0.699	0.96 [0.86, 1.07]	4.48×10^{-1}
[14]	rs4838291	9:128489937	Intergenic	G	0.392	1.08 [0.97, 1.19]	1.65×10^{-1}	0.391	1.01 [0.92, 1.11]	8.66×10^{-1}

[14]	rs112883710	9:136430088	*ADAMTSL2*	A	0.110	0.99 [0.83, 1.17]	8.85×10^{-1}	0.110	0.95 [0.80, 1.11]	5.02×10^{-1}
[14]	rs112395992	9:136449214	Intergenic	T	0.042	1.01 [0.66, 1.54]	9.75×10^{-1}	0.056	0.95 [0.65, 1.40]	8.09×10^{-1}
[14]	rs113067637	9:136452411	Intergenic	A	0.121	1.02 [0.87, 1.19]	8.33×10^{-1}	0.120	1.02 [0.88, 1.18]	7.76×10^{-1}
[14]	rs3888560	9:136465342	Intergenic	T	0.301	1.00 [0.89, 1.13]	9.55×10^{-1}	0.302	1.03 [0.92, 1.15]	6.13×10^{-1}
[14]	rs78094364	9:136466679	Intergenic	T	0.963	0.76 [0.58, 1.00]	5.19×10^{-2}	0.957	0.84 [0.66, 1.07]	1.61×10^{-1}
[14]	rs739447	9:136477511	Intergenic	T	0.135	1.02 [0.88, 1.18]	8.01×10^{-1}	0.132	0.96 [0.84, 1.11]	6.06×10^{-1}
[14]	rs7872903	9:136484292	Intergenic	C	0.251	1.04 [0.93, 1.17]	4.87×10^{-1}	0.251	0.94 [0.84, 1.04]	2.17×10^{-1}
[14]	rs1108581	9:136505241	*DBH*	A	0.809	1.08 [0.95, 1.23]	2.28×10^{-1}	0.808	0.95 [0.85, 1.07]	4.02×10^{-1}
[14]	rs10509123	10:61893145	*ANK3*	T	0.635	1.00 [0.90, 1.11]	1.00×10^{0}	0.636	0.91 [0.83, 1.00]	6.05×10^{-2}
[14]	rs7896518	10:65104500	*JMJD1C*	A	0.581	1.00 [0.91, 1.11]	9.46×10^{-1}	0.582	1.01 [0.92, 1.11]	8.66×10^{-1}
[14]	rs35649565	10:87074564	Intergenic	G	0.759	0.95 [0.85, 1.07]	3.93×10^{-1}	0.761	1.06 [0.96, 1.19]	2.58×10^{-1}
[14]	rs61861137	10:105613383	*SH3PXD2A*	A	0.759	1.01 [0.90, 1.13]	8.81×10^{-1}	0.753	0.94 [0.84, 1.05]	2.50×10^{-1}
[14]	rs1827535	11:16380409	*SOX6*	G	0.329	1.03 [0.92, 1.15]	5.93×10^{-1}	0.328	1.00 [0.90, 1.10]	9.53×10^{-1}
[14]	rs2585817	11:28602221	Intergenic	A	0.633	0.99 [0.90, 1.10]	8.82×10^{-1}	0.631	1.07 [0.97, 1.18]	1.61×10^{-1}
[14]	rs1783818	11:57437551	ZDHHC5	T	0.562	1.04 [0.94, 1.15]	4.57×10^{-1}	0.563	1.04 [0.94, 1.14]	4.64×10^{-1}
[14]	rs10792430	11:64147114	Intergenic	T	0.816	0.97 [0.86, 1.11]	6.78×10^{-1}	0.815	1.04 [0.92, 1.17]	5.48×10^{-1}
[14]	rs2734838	11:113286501	*DRD2*	A	0.407	1.05 [0.95, 1.17]	3.09×10^{-1}	0.403	1.00 [0.91, 1.10]	9.84×10^{-1}
[14]	rs11223560	11:133561399	Intergenic	G	0.418	0.98 [0.88, 1.08]	6.68×10^{-1}	0.418	0.97 [0.88, 1.06]	4.86×10^{-1}
[14]	rs11171710	12:56368078	*RAB5B*	A	0.459	1.08 [0.98, 1.19]	1.23×10^{-1}	0.459	0.97 [0.88, 1.06]	4.75×10^{-1}
[14]	rs4417327	12:58285460	*LOC283387*	C	0.563	0.96 [0.87, 1.06]	4.15×10^{-1}	0.563	0.97 [0.89, 1.07]	5.71×10^{-1}
[14]	rs8756	12:66359752	*HMGA2*	A	0.512	1.14 [1.03, 1.26]	1.12×10^{-2}	0.505	1.14 [1.04, 1.25]	5.61×10^{-3}
[14]	rs17113564	12:74904625	Intergenic	C	0.867	1.04 [0.90, 1.21]	5.68×10^{-1}	0.868	1.04 [0.91, 1.19]	5.92×10^{-1}
[14]	rs7960340	12:99737259	*ANKS1B*	A	0.142	0.92 [0.80, 1.07]	2.91×10^{-1}	0.142	0.94 [0.82, 1.07]	3.48×10^{-1}
[14]	rs678436	12:111949394	*ATXN2*	C	0.256	0.96 [0.86, 1.08]	5.42×10^{-1}	0.259	1.00 [0.89, 1.11]	9.32×10^{-1}
[14]	rs4902704	14:69703588	*EXD2*	C	0.624	0.91 [0.82, 1.00]	6.18×10^{-2}	0.625	0.94 [0.85, 1.03]	1.80×10^{-1}
[14]	rs12884296	14:79534362	*NRXN3*	A	0.386	1.01 [0.91, 1.12]	8.50×10^{-1}	0.383	1.02 [0.92, 1.12]	7.28×10^{-1}
[14]	rs7152530	14:98641215	Intergenic	A	0.363	0.96 [0.86, 1.06]	4.00×10^{-1}	0.362	0.96 [0.88, 1.06]	4.43×10^{-1}
[14]	rs34161718	14:104620193	*KIF26A*	C	0.772	0.98 [0.87, 1.10]	7.22×10^{-1}	0.768	1.02 [0.92, 1.15]	6.82×10^{-1}
[14]	rs35534970	15:47675585	*SEMA6D*	A	0.225	1.02 [0.91, 1.15]	7.43×10^{-1}	0.228	1.09 [0.98, 1.22]	1.03×10^{-1}
[14]	rs58365910	15:78849034	Intergenic	C	0.354	0.92 [0.83, 1.02]	1.17×10^{-1}	0.352	0.91 [0.82, 1.00]	5.38×10^{-2}
[14]	NA	15:78850501	Intergenic	A	0.353	0.94 [0.84, 1.05]	2.67×10^{-1}	0.349	0.90 [0.81, 1.00]	4.33×10^{-2}

[14]	rs667282	15:78863472	*CHRNA5*	T	0.790	0.98 [0.87, 1.11]	7.57×10^{-1}	0.790	0.91 [0.81, 1.02]	9.24×10^{-2}
[14]	rs150300	15:89944190	Intergenic	T	0.606	0.94 [0.84, 1.05]	2.97×10^{-1}	0.608	0.94 [0.84, 1.04]	2.48×10^{-1}
[14]	rs35198836	16:1244631	*CACNA1H*	T	0.304	0.97 [0.87, 1.08]	5.55×10^{-1}	0.303	0.94 [0.85, 1.04]	2.50×10^{-1}
[14]	rs75744751	16:53564401	Intergenic	C	0.061	1.06 [0.86, 1.31]	5.92×10^{-1}	0.061	1.11 [0.91, 1.35]	2.96×10^{-1}
[14]	rs1364349	16:61029491	Intergenic	C	0.907	0.97 [0.81, 1.15]	7.13×10^{-1}	0.905	0.88 [0.75, 1.02]	9.70×10^{-2}
[14]	rs3759982	16:69140747	*HAS3*	C	0.126	1.05 [0.90, 1.21]	5.60×10^{-1}	0.127	0.93 [0.81, 1.07]	3.34×10^{-1}
[14]	rs727428	17:7537792	Intergenic	C	0.556	0.92 [0.83, 1.02]	1.28×10^{-1}	0.559	0.97 [0.88, 1.06]	4.82×10^{-1}
[14]	rs4925130	17:17807858	*TOM1L2*	A	0.372	1.01 [0.91, 1.11]	9.14×10^{-1}	0.372	0.96 [0.87, 1.05]	3.81×10^{-1}
[14]	rs66481362	17:37758172	Intergenic	A	0.085	1.03 [0.86, 1.24]	7.54×10^{-1}	0.083	0.95 [0.80, 1.13]	5.74×10^{-1}
[14]	rs1941472	18:37702403	Intergenic	G	0.438	1.00 [0.90, 1.11]	9.87×10^{-1}	0.439	0.95 [0.86, 1.05]	3.15×10^{-1}
[14]	rs61537869	18:50542722	*DCC*	G	0.589	0.96 [0.87, 1.07]	4.56×10^{-1}	0.588	0.99 [0.90, 1.09]	8.77×10^{-1}
[14]	rs11881918	19:41334199	Intergenic	A	0.072	1.1 [0.90, 1.35]	3.34×10^{-1}	0.074	1.00 [0.83, 1.20]	9.64×10^{-1}
[14]	rs10853742	19:41340573	Intergenic	G	0.345	0.95 [0.85, 1.05]	3.23×10^{-1}	0.347	0.97 [0.87, 1.07]	4.88×10^{-1}
[14]	rs56113850	19:41353107	*CYP2A6*	T	0.432	0.91 [0.82, 1.01]	6.53×10^{-2}	0.432	0.93 [0.85, 1.02]	1.41×10^{-1}
[14]	rs28399443	19:41354306	*CYP2A6*	A	0.022	0.77 [0.53, 1.12]	1.73×10^{-1}	0.023	1.11 [0.80, 1.54]	5.35×10^{-1}
[14]	rs57837628	19:41357910	Intergenic	A	0.482	0.92 [0.83, 1.01]	9.05×10^{-2}	0.481	0.91 [0.83, 1.00]	4.18×10^{-2}
[14]	rs117824460	19:41371480	Intergenic	G	0.031	1.12 [0.81, 1.54]	4.82×10^{-1}	0.031	1.21 [0.89, 1.63]	2.19×10^{-1}
[14]	rs6011779	20:61984317	*CHRNA4*	C	0.208	1.02 [0.91, 1.16]	7.19×10^{-1}	0.206	0.90 [0.80, 1.01]	7.23×10^{-2}
[14]	rs12479680	20:61988413	*CHRNA4*	A	0.215	1.00 [0.88, 1.14]	9.86×10^{-1}	0.213	0.91 [0.80, 1.03]	1.29×10^{-1}
[14]	rs45497800	20:61991833	*CHRNA4*|*LOC100130587*	T	0.093	1.15 [0.97, 1.37]	1.13×10^{-1}	0.095	1.09 [0.93, 1.27]	3.12×10^{-1}
[14]	rs79739740	20:61992467	*CHRNA4*	T	0.089	1.15 [0.94, 1.41]	1.84×10^{-1}	0.089	1.09 [0.91, 1.32]	3.48×10^{-1}
[14]	rs11699828	20:62157198	Intergenic	G	0.970	1.09 [0.77, 1.55]	6.31×10^{-1}	0.970	1.32 [0.96, 1.83]	9.23×10^{-2}
[14]	rs9611540	22:41674203	*RANGAP1*	C	0.673	1.01 [0.91, 1.12]	8.83×10^{-1}	0.675	0.94 [0.85, 1.04]	2.50×10^{-1}
[14]	rs2542028	22:47196524	*TBC1D22A*	A	0.277	1.08 [0.96, 1.20]	1.93×10^{-1}	0.282	1.06 [0.96, 1.18]	2.43×10^{-1}

Odds ratios (ORs) and 95% confidence intervals (CIs) are aligned to the allele (EA) which increases risk of varenicline efficacy, nausea and smoking cessation.
Abbreviations: Chr, chromosome; EAF, effect allele frequency.

Supplementary Table 6 Look-up of sentinel variants associated with **(A)** SVE and **(B)** LVE in genome-wide association studies of age at smoking initiation, smoking initiation, cigarettes per day and smoking cessation performed by GSCAN[14].

Sentinel variant	Chr:Position (hg19)	EA	Age at initiation		Smoking initiation		Cigarettes per day		Smoking cessation	
			OR [95% CI]	*p*-value	OR [95% CI]	*p*-value	OR [95% CI]	*p*-value	OR [95% CI]	*p*-value
**(A) Short-term varenicline effectiveness (SVE)**										
rs186444103	2:40054022	T	0.98 [0.96, 1.00]	2.61E-02	0.99 [0.98, 1.00]	9.33E-02	0.99 [0.97, 1.01]	3.47E-01	1.00 [0.98, 1.01]	8.41E-01
rs4364036	2:162904624	T	1.00 [0.98, 1.01]	8.56E-01	1.00 [0.99, 1.01]	9.48E-01	1.01 [0.99, 1.03]	2.12E-01	1.00 [0.99, 1.02]	7.38E-01
rs4686373	3:9853437	A	1.00 [0.99, 1.01]	7.97E-01	1.00 [0.99, 1.01]	9.41E-01	1.00 [0.99, 1.01]	3.58E-01	1.00 [0.99, 1.01]	6.68E-01
rs78597169	8:117830288	A	0.99 [0.98, 1.01]	4.75E-01	1.01 [1.00, 1.03]	1.67E-02	1.00 [0.98, 1.02]	8.68E-01	1.00 [0.98, 1.01]	8.01E-01
rs2297038	10:30663464	T	1.00 [0.99, 1.01]	8.49E-01	1.00 [1.00, 1.01]	4.52E-01	1.00 [0.99, 1.01]	8.85E-01	1.01 [1.00, 1.01]	4.72E-02
rs9600669	13:76776621	A	1.00 [0.99, 1.01]	6.45E-01	1.00 [0.99, 1.00]	3.68E-01	0.99 [0.98, 1.00]	5.18E-02	1.01 [1.00, 1.02]	1.36E-02
rs8003262	14:102050501	A	1.01 [1.00, 1.02]	1.67E-02	1.00 [0.99, 1.01]	8.88E-01	1.00 [0.99, 1.01]	4.51E-01	1.00 [0.99, 1.01]	8.92E-01
rs827937	20:46793101	A	1.00 [0.99, 1.00]	2.81E-01	1.00 [0.99, 1.01]	7.90E-01	1.00 [0.99, 1.00]	1.59E-01	1.00 [0.99, 1.02]	6.36E-01
**(B) Long-term varenicline effectiveness (LVE)**										
rs895545	2:121721467	T	1.00 [0.99, 1.01]	7.16E-01	1.00 [0.99, 1.01]	8.61E-01	0.99 [0.98, 1.00]	2.36E-01	1.01 [1.00, 1.02]	1.66E-01
rs7712227	5:118656769	A	0.99 [0.98, 1.00]	1.11E-01	1.00 [0.99, 1.00]	2.14E-01	1.01 [1.00, 1.02]	1.36E-02	1.00 [0.99, 1.01]	4.10E-01
rs78695099	6:100195347	A	1.02 [1.00, 1.03]	5.87E-02	0.98 [0.97, 0.99]	1.68E-03	1.01 [0.99, 1.02]	4.71E-01	1.00 [0.98, 1.02]	9.77E-01
rs35443123	7:85173800	A	1.00 [0.99, 1.00]	3.44E-01	1.00 [1.00, 1.01]	2.36E-02	1.00 [0.99, 1.00]	1.72E-01	1.00 [0.99, 1.00]	1.15E-01
rs766169	22:47680100	T	1.00 [0.99, 1.01]	5.47E-01	1.01 [1.00, 1.02]	1.67E-02	1.00 [0.99, 1.01]	6.84E-01	1.00 [0.99, 1.01]	6.43E-01

SVE sentinels, rs149318872 and rs10421326, were not available in the GSCAN datasets and an appropriate proxy (within ±100kb with r^2 ≥0.9) could not be found. *Abbreviations:* Chr, chromosome; CI, confidence interval; EA, effect allele; OR, odds ratio.

References

1. Leitsalu L, Haller T, Esko T, et al. Cohort Profile: Estonian Biobank of the Estonian Genome Center, University of Tartu. *Int J Epidemiol*. Aug 2015;44(4):1137-47. doi:10.1093/ije/dyt268

2. John C, Reeve NF, Free RC, et al. Cohort profile: Extended Cohort for E-health, Environment and DNA (EXCEED). *Int J Epidemiol*. Oct 1 2019;48(5):1734. doi:10.1093/ije/dyz175

3. Hebert HL, Shepherd B, Milburn K, et al. Cohort Profile: Genetics of Diabetes Audit and Research in Tayside Scotland (GoDARTS). *Int J Epidemiol*. Apr 1 2018;47(2):380-381j. doi:10.1093/ije/dyx140

4. McKinstry B, Sullivan FM, Vasishta S, et al. Cohort profile: the Scottish Research register SHARE. A register of people interested in research participation linked to NHS data sets. *BMJ Open*. Feb 1 2017;7(2):e013351. doi:10.1136/bmjopen-2016-013351

5. Bycroft C, Freeman C, Petkova D, et al. The UK Biobank resource with deep phenotyping and genomic data. *Nature*. Oct 2018;562(7726):203-209. doi:10.1038/s41586-018-0579-z

6. Laisk T, Lepamets M, Koel M, Abner E, Estonian Biobank Research T, Magi R. Genome-wide association study identifies five risk loci for pernicious anemia. *Nat Commun*. Jun 18 2021;12(1):3761. doi:10.1038/s41467-021-24051-6

7. Williams AT, Chen J, Coley K, et al. Genome-wide association study of thyroid-stimulating hormone highlights new genes, pathways and associations with thyroid disease. *Nat Commun*. Oct 23 2023;14(1):6713. doi:10.1038/s41467-023-42284-5

8. Srinivasan S, Liju S, Sathish N, et al. Common and Distinct Genetic Architecture of Age at Diagnosis of Diabetes in South Indian and European Populations. *Diabetes Care*. Aug 1 2023;46(8):1515-1523. doi:10.2337/dc23-0243

9. King DP, Paciga S, Pickering E, et al. Smoking cessation pharmacogenetics: analysis of varenicline and bupropion in placebo-controlled clinical trials. *Neuropsychopharmacology*. Feb 2012;37(3):641-50. doi:10.1038/npp.2011.232

10. Chen LS, Baker TB, Miller JP, et al. Genetic Variant in CHRNA5 and Response to Varenicline and Combination Nicotine Replacement in a Randomized Placebo-Controlled Trial. *Clin Pharmacol Ther*. Dec 2020;108(6):1315-1325. doi:10.1002/cpt.1971

11. Tomaz PRX, Kajita MS, Santos JR, et al. Cytochrome P450 2A6 and 2B6 polymorphisms and smoking cessation success in patients treated with varenicline. *Eur J Clin Pharmacol*. Nov 2019;75(11):1541-1545. doi:10.1007/s00228-019-02731-z

12. Rocha Santos J, Tomaz PR, Issa JS, et al. CHRNA4 rs1044396 is associated with smoking cessation in varenicline therapy. *Front Genet*. 2015;6:46. doi:10.3389/fgene.2015.00046

13. Swan GE, Javitz HS, Jack LM, et al. Varenicline for smoking cessation: nausea severity and variation in nicotinic receptor genes. *Pharmacogenomics J*. Aug 2012;12(4):349-58. doi:10.1038/tpj.2011.19

14. Saunders GRB, Wang X, Chen F, et al. Genetic diversity fuels gene discovery for tobacco and alcohol use. *Nature*. Dec 2022;612(7941):720-724. doi:10.1038/s41586-022-05477-4
